# THE ROLE OF THE NEIGHBORHOOD-BUILT ENVIRONMENT ON OUTDOOR MOBILITY OF PEOPLE LIVING WITH DEMENTIA

**DOI:** 10.1093/geroni/igad104.0738

**Published:** 2023-12-21

**Authors:** Habib Chaudhury, Lillian Hung, Shannon Freeman, Mark Groulx, Kishore Seetharaman, Joey Wong, Cari Randa

**Affiliations:** Simon Fraser University, Vancouver, British Columbia, Canada; University of British Columbia, Vancouver, British Columbia, Canada; University of Northern British Columbia, Prince George, British Columbia, Canada; University of Northern British Columbia, Prince George, British Columbia, Canada; Simon Fraser University, Vancouver, British Columbia, Canada; University of British Columbia, Vancouver, British Columbia, Canada; Simon Fraser University, Vancouver, British Columbia, Canada

## Abstract

Dementia-friendly communities (DFCs) aim to foster a supportive, inclusive and empowering environment that promotes equal rights and resources for people living with dementia and their care partners. Central to DFCs is promoting access and navigation of outdoor spaces and destinations in the neighbourhood. While there is a growing interest and uptake of DFCs in policy and practice, there is scarce empirical evidence on the role of the built environment on mobility and navigation for people living with dementia. The “Dementia-inclusive Spaces for Community Access, Participation, and Engagement (DemSCAPE)” study aims to identify spatial and temporal patterns in activities undertaken outside home by people living with dementia, and ways in which the neighbourhood built environment affects their outdoor mobility and social participation. A series of sit-down and video-and-photo-documented walk-along interviews were conducted with 26 participants who are living with (mild to moderate) dementia or mild cognitive impairment in the Metro Vancouver region of British Columbia, Canada. Findings shed light on how people living with dementia understand and navigate the neighbourhood environment, and features that prompt recall of routes, places, and events, and support orientation and wayfinding. Findings also underscore the importance of participants’ awareness of barriers and obstacles in their neighbourhood, and how they cope with challenges and demands encountered while walking outside. The study offers planners and designers awareness and insights into the lived experience of navigating the neighbourhood environment with the condition of dementia and guidance on adopting a dementia-friendly and inclusive approach in policy and practice.

